# Real-time fMRI neurofeedback to down-regulate superior temporal gyrus activity in patients with schizophrenia and auditory hallucinations: a proof-of-concept study

**DOI:** 10.1038/s41398-017-0067-5

**Published:** 2018-02-12

**Authors:** Natasza D. Orlov, Vincent Giampietro, Owen O’Daly, Sheut-Ling Lam, Gareth J. Barker, Katya Rubia, Philip McGuire, Sukhwinder S. Shergill, Paul Allen

**Affiliations:** 1 Department of Psychosis Studies, Institute of Psychiatry, Psychology and Neuroscience, King’s College London, London, UK; 2 Department of Psychology, University of Roehampton, London, UK; 3 Department of Neuroimaging, Institute of Psychiatry, Psychology and Neuroscience, King’s College London, London, UK; 4 Department of Child and Adolescent Psychiatry, Institute of Psychiatry, Psychology and Neuroscience, King’s College London, London, UK; 5 Combined Universities Brain Imaging Centre (CUBIC), London, UK

## Abstract

Neurocognitive models and previous neuroimaging work posit that auditory verbal hallucinations (AVH) arise due to increased activity in speech-sensitive regions of the left posterior superior temporal gyrus (STG). Here, we examined if patients with schizophrenia (SCZ) and AVH could be trained to down-regulate STG activity using real-time functional magnetic resonance imaging neurofeedback (rtfMRI-NF). We also examined the effects of rtfMRI-NF training on functional connectivity between the STG and other speech and language regions. Twelve patients with SCZ and treatment-refractory AVH were recruited to participate in the study and were trained to down-regulate STG activity using rtfMRI-NF, over four MRI scanner visits during a 2-week training period. STG activity and functional connectivity were compared pre- and post-training. Patients successfully learnt to down-regulate activity in their left STG over the rtfMRI-NF training. Post- training, patients showed increased functional connectivity between the left STG, the left inferior prefrontal gyrus (IFG) and the inferior parietal gyrus. The post-training increase in functional connectivity between the left STG and IFG was associated with a reduction in AVH symptoms over the training period. The speech-sensitive region of the left STG is a suitable target region for rtfMRI-NF in patients with SCZ and treatment-refractory AVH. Successful down-regulation of left STG activity can increase functional connectivity between speech motor and perception regions. These findings suggest that patients with AVH have the ability to alter activity and connectivity in speech and language regions, and raise the possibility that rtfMRI-NF training could present a novel therapeutic intervention in SCZ.

## Introduction

Auditory verbal hallucinations (AVH) are a cardinal feature of schizophrenia (SCZ), occurring in around 70% of patients with the illness^1^. They are associated with high levels of distress as well as functional and occupational disability^2^. In 30% of patients with AVH, traditional antipsychotic drugs have little or no effect^3^. Thus, there is a clear need for an enhanced understanding of the neural systems that underlie AVH and how function within these systems can be altered.

The neural basis of AVH in patients with SCZ is not fully understood. However, over the last few decades, neuroimaging techniques have allowed researchers to identify brain regions associated with AVH^4,5^. There is now consensus that AVH are associated with a functional network of brain areas including auditory and language regions in the superior temporal gyrus (STG) and inferior parietal gyrus (IPG), and speech motor regions in the inferior prefrontal cortex/gyrus (IFG), as well as cortical midline region around the cingulate cortex and paracingulate sulcus^4,6–8^. Hyperactivity in the posterior STG, a region sensitive to speech and human voices^9^, is a particularly robust neuroimaging finding in patients with SCZ and AVH^4,7,8^. The posterior STG has been shown to spontaneously activate during silence^10^ and increased resting activity ^11^ and cerebral perfusion in this sensory region^12^ have also been reported in people with AVH. These findings are consistent with neuroimaging studies employing a “symptom-capture design” that show increased activity in the STG and temporo-parietal language regions when patients with SCZ are actively experiencing AVH^8^. Moreover, altered neuronal connectivity between STG and speech motor areas in the inferior frontal gyrus (IFG) has also been reported. Dysconnectivity between STG and speech motor regions in the IFG^13–16^ may impair the ability of the motor system to convey information about the sensory consequences of self-generated actions, such as inner speech, to sensory regions. Such a defective prediction mechanism may result in the failure of the normal attenuation of sensory response in the speech-sensitive STG to self-generated speech^14,17,18^.

Building on these neuroimaging findings, transcranial magnetic stimulation (TMS) studies have shown that a reduction in the cortical excitability of temporo-parietal sensory regions can reduce AVH severity^19,20^. Whilst this approach has the advantage of establishing causal links between these regions and AVH, it is often unattractive to patients as it involves the application of a strong magnetic pulse. Furthermore, changes in network dynamics cannot be readily investigated using TMS in isolation. Real-time functional magnetic resonance imaging neurofeedback (rtfMRI-NF) offers an alternative approach, as it can be better targeted than TMS, and it allows individuals to monitor and self-regulate their own brain activity^21,22^. During rtfMRI-NF, brain activity is measured in real time and fed back to participants so that they can progressively achieve voluntary control over their own neural activity^21^. Precisely defining the target brain area or network is important for rtfMRI-NF protocols and the vastly superior spatial resolution of MRI, as compared to alternative neurofeedback protocols using electroencephalography, allows the feedback signal to be better localized. rtfMRI-NF studies in patients with SCZ are limited, but a recent case report shows subjective improvements in some aspects of three patients’ AVH when rtfMRI-NF was applied to the anterior cingulate cortex^23^. rtfMRI-NF experiments could make a major contribution to our understanding of the brain regions and networks involved in AVH and, crucially, could enable us to test if people with SCZ can be trained to alter activity and connectivity in these networks.

The aim of the current study was to investigate, for the first time, whether patients with SCZ and AVH could learn to down-regulate left STG activity using an rtfMRI-NF training protocol. We also examined if left STG down-regulation would lead to network connectivity changes in speech motor and sensory regions. We chose to use the speech-sensitive region in the posterior STG as our rtfMRI-NF target region as it has been shown to be activated during AVH^8^, to be tonically hyperactive in patients with AVH^12^, can be functionally localized^9^, and can be effectively down-regulated by rtfMRI-NF training^24^. Changes in STG activity have also been shown to alter connections with other brain regions in healthy volunteers^25^. Specifically, we predicted that rtfMRI-NF training, targeting the voice-sensitive region of the STG, would reduce activity in this region, and enhance functional connectivity between left STG and other fronto-temporal speech and language regions. Exploratory analyzes of clinical data examined if changes in AVH symptom severity were related to neurofunctional changes.

## Methods

### Participants

Twelve right-handed participants with a Diagnostic and Statistical Manual of Mental Disorders 5th Edition^26^ diagnosis of SCZ or schizoaffective disorder were recruited to the study from the South London and Maudsely NHS Trust in London, UK. Patients were required to provide written informed consent and to be treated with stable doses of antipsychotic medication for the 3 months prior to study enrollment. Participants who met the criteria for alcohol or substance dependence in the previous 6 months were excluded. The inclusion criteria required a score of ≥3 on the hallucinatory behavior (P3) item of the Positive and Negative Syndrome Scale (PANSS)^27,28,^. The study was approved by the Stanmore National Research Ethics Committee (REC number 15/LO/1007); all study procedures have been conducted in accordance with the Declaration of Helsinki.

**Fig. 1:**
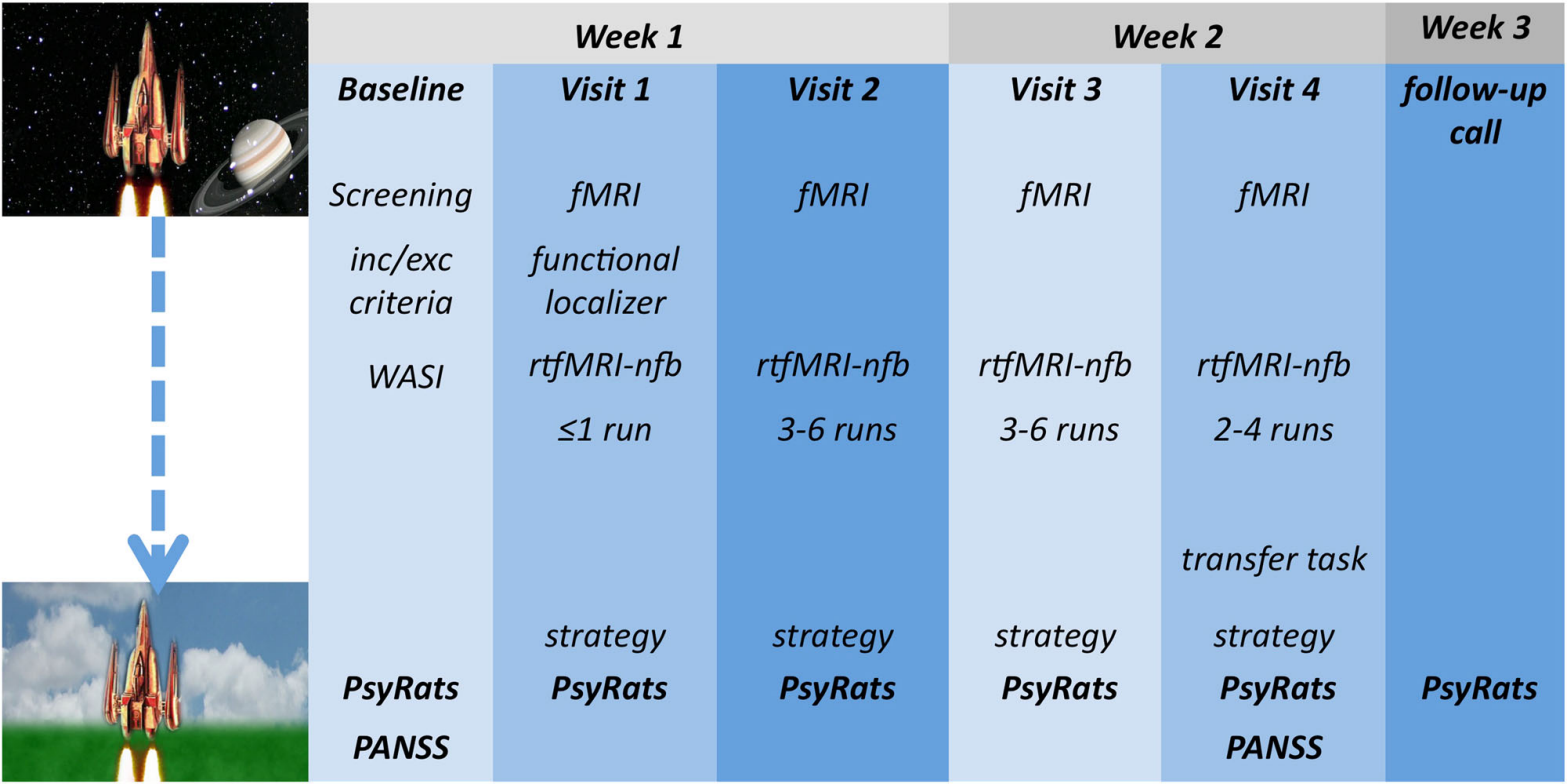
Study design. Participants attended five study visits. During the baseline visit participants were assessed on the inclusion/exclusion criteria, and clinical and socio-demographic information was collected. During the first scanning visit a mask of the speech-sensitive left STG was created for each participant using the functional localizer task: inc/exc inclusion/exclusion, WASI Wechsler Abbreviate Scale of Intelligence, PsyRats Psychotic Symptom Rating Scale, PANSS Positive and Negative Syndrome Scale, rtfMRI-NF real-time functional magnetic resonance imaging neurofeedback

Participants were required to attend the study on five separate visits (Fig. 1). Baseline clinical assessments were conducted on the first day. The four subsequent visits for MRI scans were completed over a 2-week period. Clinical assessments were also completed during these visits. Assessment included the PANSS at baseline and after the last fMRI visit, and the Psychotic Rating Symptom Scale (PsyRats)^29^ hallucination subscale at baseline, after each rtfMRI-NF scan (visits 2–4), and 1 week post fMRI.

Intelligence quotient (IQ) was assessed using subsets (Matrix reasoning, and vocabulary) of the Wechsler Abbreviated Scale of Intelligence^30^. The PANSS and PsyRats instruments were administered by an independent researcher (MS), who was blind to the study protocol and objectives.

### Imaging data acquisition

Functional images were acquired on a General Electric MR750 3.0 T MR scanner at the Department of Neuroimaging, Institute of Psychiatry, Psychology and Neuroscience, King’s College London (London, UK). A 12-channel head coil was used for radio frequency transmission and reception. A single-shot gradient recalled echo planar imaging sequence was used for fMRI acquisition (64 × 64 matrix over a 21.1 × 21.1 cm^2^ field of view (FOV), giving an in-plane voxel size of 3.3 mm; slice thickness 3 mm with a 0.3 mm slice gap; repetition time (TR) = 2000ms; echo time (TE) = 30ms, flip angle = 75°). A high-resolution three-dimensional (3D) T1-weighted enhanced gradient echo (256 × 256 matrix over a 27 × 27 cm^2^ FOV, giving an in-plane voxel size of 1.05 mm; slice thickness 1.2 mm; TR = 7.312 ms, TE = 3.015 ms, flip angle = 11°) scan was acquired for image normalization. The first four volumes of each rtfMRI-NF run were discarded to allow steady-state magnetization to be established.

### Localizer scan

During the first visit participants underwent a localizer scan to identify voice-sensitive regions in their left STG. This consisted of a voice perception task comprising blocks of vocal and non-vocal stimuli^9^. Vocal stimuli were words for everyday objects neutral in semantic and prosodic content, whereas the non-vocal stimuli were based on non-speech digitalized sounds with amplitude and energy matched with control sounds. The presentation was alternated, with a total of eight 20 s blocks of vocal and seven 20 s blocks of non-vocal stimuli, lasting approximately 5 min in total. Stimuli loudness was adjusted individually, to ensure that participants could hear the stimuli clearly above the scanner noise. To create a functional mask from which to derive the rtfMRI-NF signal, we calculated the effective signal change in areas activated by the functional localizer task using conventional univariate fMRI analysis techniques. This was based on the task contrast of the average BOLD signal between the activation block (vocal stimuli) and the baseline block (non-vocal stimuli). Online pre-processing and analysis were conducted using AFNI software (https://afni.nimh.nih.gov/) and using local scripts written and developed by the author V.G. In short, the data were smoothed and corrected for head motion. The contrast vocal > non-vocal was chosen and faces touching clusters with the highest t-statistic were displayed (https://afni.nimh.nih.gov/pub/dist/doc/program_help/3dclust.html). The individual functional masks were created based on the maximally activated cluster in the left posterior STG by manually thresholding the target cluster until the required size/shape was present in the left posterior STG (ROI_STG_). The left STG was chosen because this region is typically active during AVH. We used white matter as a reference region to cancel out non-specific global brain effects (ROI_REF_): a white matter mask was created by segmenting the T1-weighted structural image in AFNI, eroding to limit partial volume effects, and mapping it onto the functional localizer mask by reversing the normalization process.

### rtfMRI-NF data acquisition and processing

A custom rtfMRI-NF interface system^31^ and AFNI software^32^ were used for real-time transfer and analysis of fMRI data. The rtfMRI-NF interface system ran on the scanner hardware to access the fMRI scans as they were reconstructed. The images were then transferred to a Linux workstation where they were pre-processed using AFNI’s built in real-time capabilities. Once the target in the STG ROI (ROI_STG_) had been identified using the functional localizer described above, the neurofeedback signal was calculated using the formula: ((ROI_STG_−ROI_REF_)−(ROI _STG_ Previous−ROI_REF_ Previous)), where the previous ROIs are the average activation of the left STG and references regions in the previous rest block. Thus, the NF signal was a function of the difference of the current ROI_STG_ activity (averaged over 3 TR periods in order to reduce jitter) to the average of the previous rest block, with all values being measured relative to the corresponding white matter signal (changes which represent global signal variations of no interest).

### rtfMRI-NF training

Each rtfMRI-NF training run comprised a block design similar to that used in previous studies^24,33^. Each rtfMRI-NF training run alternated between no-regulation “rest” blocks (7 blocks of 30 s) and down-regulation blocks (6 blocks of 50 s), lasting around 9 min per run. To motivate participants during training runs, and to provide a more visually engaging task, we used a visual feedback interface depicting a “vertically orientated space rocket”^33^. Participants were instructed to land the rocket by bringing it down to Earth. Visual feedback was provided during the training blocks, and during the rest block participants observed a fixation cross. After each visit participants reported the strategy that they used to down-regulate their left STG activity. Participants were informed about the inherent delay in feedback due to the hemodynamic response (approx. 6 s). To enhance motivation and the likelihood of successful left STG signal down-regulations, we did not provide any overt instructions or suggest any strategies; participants were asked to devise their own strategy to down-regulate their left STG signal^34,35^ (Supplementary material). All participants attended four 1-h visits for MRI. The functional localizer task was completed during the first scanner visit. During visits 2, 3, and 4, participants completed between 2 and 6 rtfMRI-NF training runs depending on the time available (mean number of runs per scanner v2 = 4.8, v3 = 4.5, v4 = 3.6). During the fourth (final) visit, participants also undertook a “transfer” run. The transfer run was identical to the training runs except that no visual feedback (static picture) was given. This allowed the overall success of the training to be assessed (i.e., participants’ ability to down-regulate their STG signal in the absence of direct feedback). Participants were informed that the picture would remain static, and were asked to employ the same strategies they used during the rtfMRI-NF training. Transfer runs measure retention of learning and are considered a proximal measure of successful transfer of training strategies to everyday life.

### Data analysis

#### Clinical data

To assess overall clinical change, and any adverse effect of the study procedures on clinical presentation, we conducted a paired *t* test for PANSS scores pre and post rtfMRI-NF training. To investigate the specific change in AVH symptoms over the rtfMRI-NF training period, we analyzed the total PsyRats AVH symptoms score by specifying a full maximum-likelihood random-effect multilevel model (MLREM)^36^. Post hoc exploratory analyses examined changes over the rtfMRI-NF training period in individual PsyRats items using one-sided, paired-sample *t* tests. These results are reported at an uncorrected threshold of *p* < 0.05, but no tests survived correction for multiple testing (*p* = 0.05/11). Data distribution checks and statistical analyses were carried out using STATA 12.1.

#### fMRI data analysis

All offline data were pre-processed and analyzed using Statistical Parametric Mapping 12 (SPM12). All functional data were slice-timed corrected and realigned, to correct for volume-to-volume head motion. Following this, the time series was co-registered to the high-resolution T1-weighted image, and normalized into the Montreal Neurological Institute (MNI) template using parameters generated by unified segmentation of the T1-weighted structural image^37^. The transformed data were smoothed using an 8 mm full-width at half-maximum isotropic Gaussian kernel. For the localizer task subject-specific fixed models were constructed with regressors encoding the predicted blood oxygenation level-dependent (BOLD) response for vocal stimuli and non-vocal stimuli blocks. For the rtfMRI-NF runs, subject-specific fixed-effects models were constructed with regressors encoding the predicted BOLD response for each of the rtfMRI-NF runs (ranging from 9 to 16 across subjects), with baseline (rest) blocks serving as the baseline. For both the localizer task and the rtfMRI-NF runs, the six motion parameters for each run, generated during realignment, were included as nuisance regressors. For all first-level models voxelwise parameter estimates for these regressors were obtained by restricted maximum-likelihood estimation using a temporal high-pass filter (cutoff = 128 s) to remove low-frequency drifts, and modeling temporal autocorrelation across scans with an AR(1) process. Following parameter estimation, contrasts of beta coefficients for the primary contrasts of interest were generated. For the localizer task the contrast was vocal stimuli > non-vocal stimuli. As the number of rtfMRI-NF runs differed between participants (9–16; mean = 14; median = 14), the second-level model comprised contrasts for the first vs. last rtfMRI-NF run acquired during the second, third, and fourth scanner visits, entered into a repeated-measures analysis of variance (ANOVA) (i.e., 6 contrasts). Finally, to check that down-regulation effects in the left STG were not due to repeated exposure to the rtfMRI-NF task (i.e., habituation) over runs within a single visit, we examined the main effect of “visit” as this would be less prone to habituation confounds, that is, patients would be unlikely to show habituation in the left STG from visit to visit.

#### Transfer task

Using a separate model, subject-specific first-level models were created using a regressor encoding the predicted response for the first rtfMRI-NF run vs. the transfer run. Motion parameters generated during the realignment for both runs were also included. A second-level paired *t* test was used to test for effects in the left STG. The association between changes in left STG activity and changes in PsyRats total scores was investigated using a regression model with a single contrast image (first > transfer run) from each subject and their corresponding change in PsyRats scores as a regressor.

**Fig. 2:**
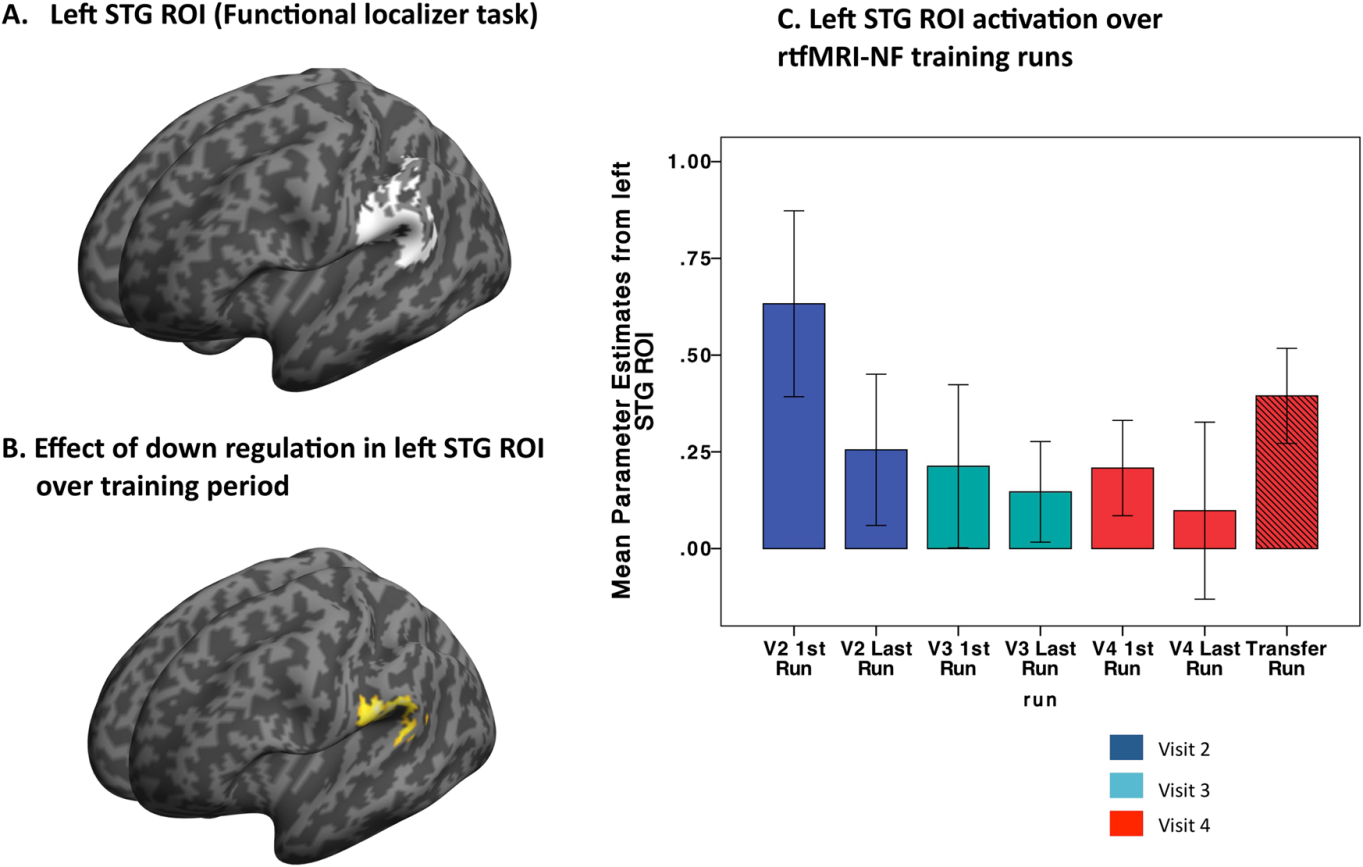
**a** 3D SPM render of mean STG ROI based on localizer task (vocal stimuli > non-vocal stimuli). **b** 3D SPM render showing effect of rtfMRI-NF training in left STG ROI. **c** Plot showing effect of rtfMRI-NF training in left STG ROI (first/last run from visits 2, 3, and 4 plus transfer scan (visit 4)

In order to focus on signal change in voice-sensitive regions of the left STG, we conducted analyses within the mean left STG ROI created by the localizer task (contrast: vocal stimuli > non-vocal stimuli). First, we adjusted obliqueness to match the individual T1-weighted structural image, removed the white matter control regions, and transformed the individual masks into MNI space, using parameters generated by unified segmentation of the T1 image. The mean mask was then computed using ImCalc in SPM12 based on the transformed individual functional localizer masks (Fig. 2a). For all second-level analyses, significant ROI results are reported at a *p* value of ≤0.05 following family-wise error correction on the basis of response amplitude (i.e., peak-level family-wise error (FWE)).

#### Psychophysiological interactions

The effect of rtfMRI-NF training on functional connectivity was investigated by a PPI analysis with a left STG seed region derived from the group ANOVA analysis. First, the eigenvariate from the seed region extracted from the subject-specific models described above. We used a 6 mm radius sphere, to pin the PPI analysis seed within the cluster size obtained in the repeated-measures ANOVA. Subject-specific response peak were required to be within the 6 mm of the group peak to be included in the PPI analysis. This was the case for all participants. Subsequently, two PPI regressors were created via deconvolution of the eigenvariate time series and by weighting of the resultant time series with the task contrast time series (the first and last rtfMRI-NF run). The regressors were adjusted for the effect of interest, and reconvoled with the hemodynamic response function to provide the predicted temporal pattern of BOLD signal that would be expected in a region if its connectivity was changed when the seed is modulated by the rtfMRI-NF run (i.e., first vs. last). The resulting two contrasts (from the first and last rtfMRI-NF runs) were tested with a paired-sample *t* test at the second-level analysis. Given our interest in functional connectivity changes in frontal and temporal speech and language regions activated during AVH, we studied changes in two ROIs taken from a meta-analysis of functional imaging studies in patients with AVH. An IFG (Broca’s area) ROI was specified using MNI coordinates (−48, 10, 8) and an ROI in the supramarginal gyrus was specified using (−52, −20, 15). Associations between left STG ROI functional connectivity and changes in PsyRats total scores were investigated using a regression model. PPI results are reported at a *p* value of ≤0.025 following FWE_peak_ and correction for two ROIs.

## Results

### Patient characteristics and symptoms

Patients’ clinical and socio-demographic information characteristics and IQ are reported in Table 1. One participant’s data was excluded due to excessive movements during the fMRI acquisition. Therefore, data in 11 participants were analyzed. rtfMRI-NF training did not worsen clinical symptoms as assessed by the PANSS scores (pre vs. post training scores) (Table 2.)

**Table 1:** Socio-demographic information of study participants

*N* = 11	Mean	SD
Age	35.1	8.1
Gender	2 Females	
Education (years)	11.9	1.5
Duration of illness (years)	10.8	8.4
Age at onset (years)	24.3	7
Medication* mg	607.5	330
WASI IQ	98	18.7

*Chlorpromazine equivalent; *WASI* Wechsler Abbreviated Scale of Intelligence

**Table 2:** PANSS and PsyRats pre vs. post training; *N* = 11

**Pre rtfMRI-NF**	**Mean**	**SD**	**Post rtfMRI-NF**	**Mean**	**SD**	**Sig**	**Test statistic**	**Effect size**
PANSS Pos.	20.4	5.5	PANSS Pos.	21.3	5.5	0.32	*t* = 1.05	
PANSS Neg.	13.5	4.9	PANSS Neg.	14.5	5.3	0.72	*t* = 0.38	
PANSS Gen.	35.2	8.9	PANSS Gen.	34.6	7.6	0.4	*t *= −0.89	
PsyRats Tot	25.2	4.4	PsyRats Tot	22.1	8	0.09	*t* = 1.38	Cohen’s *d* = 0.45
PsyRats BAO	2.7	1.1	PsyRats BAO	2	1.2	*0.04*	*t* = 1.91	Cohen’s *d* = 0.65
PsyRats IOD	2.6	1	PsyRats IOD	1.9	1.1	*0.02*	*t* = 2.34	Cohen’s *d* = 0.66

*PANSS* Positive and Negative Syndrome Scale, *Pos.* positive subscale, *Neg*. negative, *Gen*. general psychopathology subscale, *PsyRats* Psychotic Symptom Rating Scale, *Tot* Total, *BAO* beliefs about origins, *IOD* intensity of distress*, italics* statistical significance

There was a trend effect for reduced PsyRats Total scores pre vs. post rtfMRI-NF training (Table 2). MLREM revealed a significant change in PsyRats Total scores over the rtfMRI-NF training period *p = *0.015; (*b = −*0.76, 95% confidence interval: −1.38 to 0.15). Exploratory analysis of PsyRats sub-items showed that there was a post training reduction in scores for PsyRats item 5 (beliefs regarding the origin of voices) (*p* = 0.05, *t *= 1.91) and PsyRats Item 9 (Intensity of distress) (*p *= 0.02, *t = *2.34). The post training effect for item 5 was maintained at a trend level (*p *= 0.055, *t *= 1.77*)* at 1-week follow-up. However, single PsyRats item effects did not survive correction for multiple testing.

### Localizer scan

Across all participants the functional localizer task activated the left STG. Compared to non-vocal stimuli, vocal stimuli activated the voice-sensitive posterior left STG (*x*, *y*, *z* = −64, −14, 4); (*t*_1(10)_ = 3.16 [*t*_peak_ = 5.76] *K*_E_ *=* *294*, *P*_FWE_ *=* 0.02, *z*-score_peak_ = 3.74) with a sub-peak at (*x*, *y*, *z* = −64, −26, −4); ([*t*_peak_ *=* 5.59], *P*_FWE_ *=* 0.02, *z*-score_peak_ *=* 3.68) (Fig. 2a).

### rtfMRI-NF training

There was a significant decrease in activity over rtfMRI-NF training runs in a cluster of *K*_E_ = 405 in the left STG/superior temporal sulcus (*x*, *y*, *z = *−60, −30, 20); (*t*_1(60)_ = 2.39 [*t*_*peak*_* = 4.22*], *P*_FWE_* = *0.013, *z*-score_peak_ = 3.93), extending to the supramarginal gyrus/IPG (*x*, *y*, *z* = −58, −36, 24); (*t*_1(60)__ =_ 2.39 [*t*_*peak*_ = 3.48], *P*_FWE_ *=* 0.09, *z*-score_peak_ = 3.31) (Fig. 2b, c). There were no regions within the left STG ROI that showed increased activation over the course of the rtfMRI-NF. The down-regulation effect in the left STG ROI was significant across visits 2, 3, and 4 (*x*, *y*, *z* = −60, −28, 20); (*t*_1(60)__ = _2.39[*t*_*peak*_ = 4.26], *P*_FWE_ = 0.011, *z-score*_peak_ = 3.96). Over the rtfMRI-NF training protocol, there was no suprathreshold effect during the baseline blocks, that is, STG ROI activity during baseline blocks remained stable across the training protocol.

### Transfer task

Relative to the first rtfMRI-NF training run, there was a significant reduction in activity in the left STG ROI during the transfer run *x*, *y*, *z* = (−60, −28, 16); (*t*_1(10) = _2.76[*t*_peak_ = 6.59]; *K*_E_ = 156, *P*_FWE_ = 0.03, *z*-score_peak_ = 3.99).

### PPI analysis

**Fig. 3:**
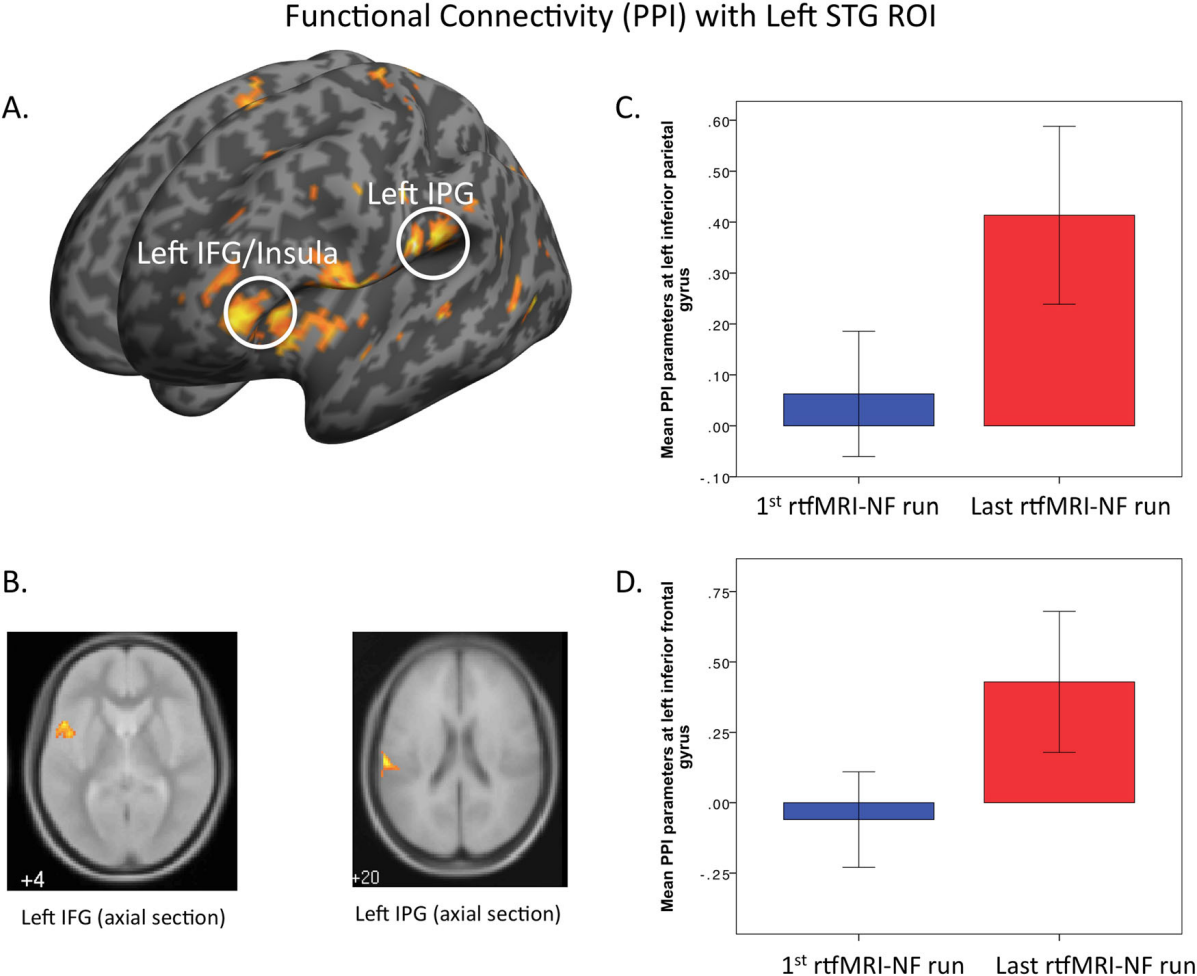
**a** SPM 3D render showing regions in left inferior frontal gyrus (IFG)/Insula and left inferior parietal gyrus (IPG)/supramarginal gyrus where functional connectivity was increased in the last rtfMRI-NF run compared to the first rtfMRI-NF (left hemisphere shown on left side of image) (*p *= 0.001 *unc* for purpose of figure). **b** Axial volumes showing left IFG and IPG regions. **c** Mean PPI parameter estimates in IPG/supramarginal gyrus for 1st vs. last rtfMRI-NF run and **d** mean PPI parameter estimates left insula/IFG for 1st vs. last rtfMRI-NF run

Using the seed region in the left posterior STG, PPI analysis revealed greater functional connectivity parameter estimates during the last rtfMRI-NF, relative to the first rtfMRI-NF run in a cluster covering the left insula extending to the left IFG (*x*, *y*, *z* = −48, 10, 2) (*t*_1(10)__ = _6.66 [*t*_*peak*_ = 6.38]; *K*_E_ = 34, *P*_FWE_* =* 0.002, *z-* score_peak_ = 3.94) and in a cluster in the supramarginal gyrus/IPG (*x*, *y*, *z*, = −52, −20, 16), (*t*_1(10)_ = 4.14 [*t*_peak_ = 5.11]; *K*_E_* = 81*, *P*_FWE_ = 0.011, *z*-score_peak_ = 3.51) (Fig. 3b). There were no areas where functional connectivity decreased after rtfMRI-NF training.

### PsyRat associations

A regression model testing the association between left STG down-regulation during the transfer run (1st run vs. transfer run) and changes in PsyRats Total scores was non-significant. There was, however, a significant positive association between change in PsyRat Total scores over the rtfMRI-NF training period (pre–post training scores) and functional connectivity between the left STG seed region and the left insula/IFG during the last rtfMRI-NF run (*x*, *y*, *x* = −48, 12, 4); (*t*_1(09)_ = 1.83 [t_*peak*_ = 6.77]; *K*_E = _233, *P*_FWE = _0.01, *z*-score_peak_ = 3.90).

## Discussion

We showed in this pioneering study that patients with SCZ and AVH could learn to down-regulate activity in speech-sensitive regions of their left STG with an average of 14 runs of 9 min NF training. Left STG down-regulation was seen between scanner visits suggesting that this effect could not easily be attributed to within visit STG habituation over rtfMRI-training runs. Furthermore, rtfMRI-NF training resulted in increased functional connectivity between frontal and temporal language regions that was associated with changes in AVH symptom severity. During the transfer scan, patients were able to reduce activity without visually presented feedback, suggesting that they had learnt strategies to reduce activity in the voice-sensitive region of the left STG.

It has been reported that AVH are associated with spontaneous brain activity^10^, increase in blood flow^12^, and increased resting state activity in the posterior STG^11^. Reduced activity in the left STG brought about by rtfMRI-NF training demonstrates that participants can develop and apply an endogenous technique to regulate brain activity in this region, known to be involved in linguistic perception^5^, and active when patients with SCZ are experiencing AVH^4^. However, although activity in the posterior STG region appears to be central to the perceptual experience, dysfunction in non-sensory regions is also likely to contribute to the phenomenological characteristics of AVH^4,5,38^. Several studies have reported altered functional^13,15,16,39^ and structural connectivity^40^ in fronto-temporal and perisylvian language networks. In the present study, the enhanced coupling between the posterior STG seed region and the insula/IFG brought about by rtfMRI-NF training is of particular interest. The IFG region contains the speech motor area (i.e., Broca’s area), also reported to be active during AVH^4^. Moreover, the subjective reality of AVH is associated with increased activity in the IFG^41^, a region involved in both inner speech and in the imagination of others’ speech^42–44^. Inner-speech models of AVH posit that increased activity in speech perception regions in the posterior STG is a consequence of reduced or defective signaling from speech motor areas; that is, speech motor regions fail to attenuate sensory activation in the STG^14,45,46^. Consequently, this failure can produce confusion regarding the agency between one’s own actions/inner-speech and externally generated actions such as perceived voices and speech^47^. The finding of increased coupling between the posterior STG region and the left insula (part of the same IFG cluster) is also of interest. This region is thought to be an extension of Broca’s convolution important for verbal imagery^48^ and volumetric change in the insula has been shown to be correlated with the intensity of AVH^49^. Functional connectivity analysis also revealed increased coupling between the posterior STG seed region and a cluster just superior to the seed region in the left IPG/supramarginal gyrus, which is part of Wernicke’s area, and is known to be involved in speech processing^50^. Reduced white matter integrity between left posterior STG and IPG has been reported previously in SCZ patients with AVH^40^, suggesting reduced connectivity *within* speech and language perception areas, as well as with more distal speech motor regions in the IFG. A recent functional imaging study in SCZ patients with AVH, reports that patients displayed a deficit in prediction errors signaling in the posterior STG; the same region activated when patients experience AVH^51^. It is possible that sensory hyperactivity in speech perception regions may arise through sensory learning and predictive coding deficits within these regions, influencing prior beliefs on sensory input. Such a learning mechanism may rely on efficient coupling within speech and language regions.

Overall, rtfMRI-NF training reduced activity in the posterior, speech-sensitive, STG, resulting in increased coupling between inferior frontal and posterior temporal speech and language regions. These findings are broadly consistent with TMS studies that report a reduction in AVH symptomatology after low-frequency TMS is used to reduce cerebral perfusion in the STG, IFG (and cingulate cortex) measured with arterial spin labeling^52^. However, compared to rTMS, rtfMRI-NF protocols can be more targeted, helping to establish causality of associations between functional and clinical phenotypes. The study has some limitations that should be discussed. We did not combine our functional localizer with an anatomical mask, which would help to define the left STG. However, as can been seen Fig. 2a, our task isolated the left STG and contiguous inferior parietal cortex, that is, brain regions associated with the speech-sensitive cortex and regions that are associated with AVH. Further, the sample size was small and we did not compare our experimental group to a control condition (e.g., a sham rtfMRI-NF condition). Thus, it cannot be ruled out that the reduced left STG activity observed over the training period was a placebo effect of some kind. We will now need to conduct a larger randomized control trial to exclude this possibility and to test if rtfMRI-NF training can be used as a novel therapeutic intervention in people with SCZ and AVH. It does seem, however, that patients with SCZ and AVH do have the ability to regulate activity (neural plasticity, i.e., alter the functional organization of the brain) in speech sensory–motor regions, in contrast to suggestions that patients have a general deficit in cortical plasticity^53^. This may open the way for wider investigations using neurotherapies, such as rtfMRI-NF, in people with SCZ.

## Electronic supplementary material

Supplementary material
