# Supplementary material for: Real-time fMRI neurofeedback to down-regulate superior temporal gyrus activity in patients with schizophrenia and auditory hallucinations: a proof-of-concept study
Source: Transl Psychiatry. 2018 Feb 12;8:46. doi: 10.1038/s41398-017-0067-5 (PMC5865171; doi:10.1038/s41398-017-0067-5)

**Supplementary material:**

**Methods:**

After each of the scanning visits participants were asked about their rtfMRI-NF scan and the strategy they applied to move the rocket. In addition, participants shared their study experience after the last rtfMRI-NF scan (see Appendix). The responses were analysed using a general inductive approach to qualitative data analysis proposed by Thomas [^1^](#_ENREF_1). The method consists of reading and reviewing the data multiple times to extract common themes to be evaluated and interpreted within the context of the study.

**Results:**

Table 1. Decreases in neuornal activity in individual study participants after rtfMRI-NF based on the individiual functional localiser masks.

| **Participant** | **coordinates** | | | **cluster** | **peak** | **peak** | **peak** | **peak** |
| --- | --- | --- | --- | --- | --- | --- | --- | --- |
|  | **x** | **y** | **z** | **size** | **T** | **Z** | **p<0.005** | **FWE** |
| **1** | **-56** | **-22** | **0** | **666** | **4.32** | **4.31** | **0.000** | **0.002** |
|  | **-48** | **-36** | **8** |  | **3.51** | **3.51** | **0.000** | **0.03** |
|  | -46 | -38 | 4 |  | 3.21 | 3.2 | 0.001 | ns |
|  | -60 | -4 | -4 |  | 3.1 | 3.1 | 0.001 | ns |
|  | -52 | -12 | 12 |  | 2.76 | 2.76 | 0.003 | ns |
|  | -50 | -46 | 12 |  | 2.49 | 2.49 | 0.006 | ns |
|  | -48 | -30 | -2 |  | 2.25 | 2.25 | 0.012 | ns |
|  | -62 | -44 | 0 |  | 2.11 | 2.11 | 0.017 | ns |
|  | -66 | -26 | 2 |  | 1.93 | 1.93 | 0.026 | ns |
|  | -58 | -46 | 12 |  | 1.93 | 1.93 | 0.027 | ns |
|  | -64 | -48 | 12 | 4 | 2.1 | 2.1 | 0.018 | ns |
| **2** | -54 | 4 | 0 | 41 | 2.55 | 2.55 | 0.005 | ns |
|  | -60 | -30 | 18 | 17 | 2.26 | 2.26 | 0.012 | ns |
|  | -62 | -20 | 18 |  | 2.2 | 2.21 | 0.014 | ns |
|  | -64 | -38 | 10 | 11 | 1.84 | 1.85 | 0.032 | ns |
|  | -52 | -22 | 14 | 1 | 1.67 | 1.68 | 0.047 | ns |
| **3** | -54 | -30 | 26 | 9 | 2.22 | 2.22 | 0.013 | ns |
|  | -52 | -30 | 14 | 20 | 2.17 | 2.17 | 0.015 | ns |
|  | -60 | -48 | 8 | 3 | 1.95 | 1.95 | 0.025 | ns |
| **5** | **-42** | **-72** | **4** | 233 | **5.59** | **5.57** | **0.000** | **0** |
|  | **-48** | **-74** | **4** |  | **4.62** | **4.61** | **0.000** | **0** |
|  | **-48** | **-76** | **0** |  | **4.45** | **4.44** | **0.000** | **0.001** |
|  | **-48** | **-80** | **-8** |  | **3.98** | **3.97** | **0.000** | **0.004** |
|  | -52 | -72 | -6 |  | 2.93 | 2.92 | 0.002 | ns |
|  | -48 | -72 | -10 |  | 2.6 | 2.6 | 0.005 | ns |
|  | -60 | -62 | -2 | 132 | 3.14 | 3.14 | 0.001 | ns |
|  | -60 | -56 | 0 |  | 2.99 | 2.98 | 0.001 | ns |
|  | -52 | -42 | 6 |  | 2.82 | 2.82 | 0.002 | ns |
|  | -64 | -52 | -4 |  | 2.76 | 2.75 | 0.003 | ns |
|  | -56 | -46 | 14 |  | 2.24 | 2.24 | 0.012 | ns |
|  | -52 | -48 | 10 |  | 2.22 | 2.22 | 0.013 | ns |
|  | -66 | -40 | 10 | 17 | 2.53 | 2.53 | 0.006 | ns |
|  | -66 | -34 | 8 |  | 2.52 | 2.52 | 0.006 | ns |
|  | -64 | -28 | 4 | 8 | 2.14 | 2.14 | 0.016 | ns |
|  | -64 | -24 | 2 |  | 1.96 | 1.97 | 0.025 | ns |
|  | -50 | -64 | 14 | 8 | 1.95 | 1.95 | 0.025 | ns |
|  | -52 | -54 | 16 | 2 | 1.79 | 1.79 | 0.036 | ns |
|  | -58 | -64 | 2 | 1 | 1.72 | 1.73 | 0.042 | ns |
|  | -52 | -36 | 2 | 1 | 1.66 | 1.67 | 0.047 | ns |
| **6** | **-54** | **-46** | **16** | 562 | **6.34** | **6.33** | **0.000** | **0** |
|  | **-50** | **-44** | **12** |  | **6.23** | **6.22** | **0.000** | **0** |
|  | **-48** | **-46** | **4** |  | **4.6** | **4.59** | **0.000** | **0** |
|  | -48 | -42 | -6 |  | 3.07 | 3.06 | 0.001 | ns |
|  | -58 | -42 | 0 |  | 2.91 | 2.91 | 0.002 | ns |
|  | -64 | -28 | -6 |  | 2.85 | 2.85 | 0.002 | ns |
|  | -60 | -34 | -6 |  | 2.85 | 2.85 | 0.002 | ns |
|  | -52 | -44 | -6 |  | 2.81 | 2.81 | 0.002 | ns |
|  | -62 | -44 | 4 |  | 2.7 | 2.7 | 0.003 | ns |
|  | -54 | -30 | -8 |  | 2.64 | 2.64 | 0.004 | ns |
|  | **-60** | **-6** | **2** | 89 | **3.99** | **3.98** | **0.000** | **0.006** |
|  | **-58** | **-24** | **14** | 5 | **3.43** | **3.43** | **0.000** | **0.035** |
|  | -64 | -18 | 10 | 8 | 2.26 | 2.26 | 0.012 | ns |
| **7** |  |  |  |  |  |  | ns | ns |
| **8** | **-52** | **0** | **2** | 146 | **3.24** | **3.23** | **0.001** | **0.048** |
|  | -50 | 10 | 0 |  | 2.75 | 2.74 | 0.003 | ns |
|  | -56 | -30 | 12 | 5 | 1.81 | 1.82 | 0.034 | ns |
|  | -58 | -4 | 0 | 1 | 1.8 | 1.81 | 0.035 | ns |
|  | -42 | -16 | 6 | 11 | 1.75 | 1.76 | 0.039 | ns |
| **9** | **-64** | **-50** | **12** | 214 | **3.72** | **3.72** | **0.000** | **0.015** |
|  | -52 | -50 | 26 |  | 3.29 | 3.29 | 0.000 | ns |
|  | -52 | -46 | 30 |  | 2.47 | 2.47 | 0.007 | ns |
|  | -52 | -8 | -10 | 26 | 3.02 | 3.02 | 0.001 | ns |
|  | -62 | -10 | -2 | 22 | 2.26 | 2.26 | 0.012 | ns |
|  | -52 | 2 | -6 | 2 | 1.79 | 1.8 | 0.036 | ns |
| **10** | **-54** | **-36** | **-4** | 903 | **6.42** | **6.4** | **0.000** | **0** |
|  | **-60** | **-60** | **0** |  | **5.97** | **5.96** | **0.000** | **0** |
|  | **-52** | **-48** | **-4** |  | **5.53** | **5.52** | **0.000** | **0** |
|  | **-64** | **-42** | **4** |  | **5.3** | **5.29** | **0.000** | **0** |
|  | **-62** | **-36** | **14** |  | **4.42** | **4.42** | **0.000** | **0.001** |
|  | **-60** | **-30** | **16** |  | **3.88** | **3.87** | **0.000** | **0.008** |
|  | -52 | -28 | 14 |  | 2.37 | 2.37 | 0.009 | ns |
|  | -54 | -12 | -8 | 4 | 2.54 | 2.54 | 0.006 | ns |
| **11** | **-64** | **-22** | **12** | 257 | **3.58** | **3.58** | **0.000** | **0.023** |
|  | -52 | -12 | 8 |  | 2.88 | 2.88 | 0.002 | ns |
|  | -50 | -4 | 6 |  | 2.76 | 2.76 | 0.003 | ns |
|  | -60 | 0 | 4 |  | 2.46 | 2.46 | 0.007 | ns |
|  | -56 | 4 | -4 |  | 1.83 | 1.84 | 0.033 | ns |
|  | -64 | -8 | -10 | 22 | 2.94 | 2.94 | 0.002 | ns |
|  | -56 | -4 | -10 |  | 2.53 | 2.53 | 0.006 | ns |
|  | -62 | -60 | -12 | 27 | 2.56 | 2.56 | 0.005 | ns |
|  | -64 | -54 | -18 |  | 2.25 | 2.25 | 0.012 | ns |
|  | -66 | -48 | -14 |  | 2.07 | 2.07 | 0.019 | ns |
|  | -62 | -26 | -6 | 21 | 2.16 | 2.16 | 0.015 | ns |
|  | -56 | 0 | -8 | 1 | 1.8 | 1.81 | 0.035 | ns |
|  | -58 | -48 | -12 | 1 | 1.75 | 1.76 | 0.039 | ns |
| **12** | **-44** | **-52** | **14** | 28 | **4.46** | **4.45** | **0.000** | **0.001** |
|  | **-44** | **-54** | **8** |  | **3.57** | **3.57** | **0.000** | **0.023** |
|  | -58 | 2 | -2 | 60 | 2.83 | 2.83 | 0.002 | ns |
|  | -62 | -8 | -6 |  | 1.82 | 1.82 | 0.034 | ns |
|  | -58 | -6 | -6 |  | 1.78 | 1.79 | 0.037 | ns |
|  | -62 | -10 | 6 | 53 | 2.29 | 2.29 | 0.011 | ns |
|  | -56 | -22 | 12 |  | 2.27 | 2.27 | 0.012 | ns |
|  | -60 | -36 | -8 | 4 | 1.94 | 1.95 | 0.026 | ns |
| FWE: family wise error; ns: not significant | | | | | | | | |
|  |  |  |  |  |  |  |  |  |

Thoughts on the scanning visit:

The analysis revealed five common themes which were *neutral, enjoyable, tiring, challenging and relaxing*. All of the participants described the rtfMRI-NF procedure using neutral adjective. For example, the majority of participants used the word ‘fine’ and ‘okay’ to describe the procedure. Six of the participants added that they found the rtfMRI-NF procedure enjoyable (i.e. *fun, interesting, good, cool, happy and pleasant*). However, six of the participant reported the training to be tiring (i.e. *draining, almost fell asleep, headache*). Five out of twelve participants also found the scanning sessions *relaxing*, and three out of twelve participants described the rtfMRI-NF sessions as challenging (*hard, not easy, tough*).

Strategies used:

The analysis of the raw data revealed the most common strategy was based on focus and concentration (i.e. *‘I concentrated’, or ‘I concentrated on flying the rocket’*). Five out of twelve participants suggested that they did not employ a specific strategy, had forgotten and/or could not come up with a strategy for some of the visits; *‘no particular single strategy, some memories, can’t concentrate on a lot of incoming thoughts’, ‘I did it with my mind’*.

Thoughts on the study

The analysis of the raw responses revealed that elven of twelve participants found partaking in the study useful (question 3). Eight of the participants reported that they had learned something useful, and half of the participants suggested that they would be able to apply the learned strategies outside of the study; i.e. (‘*Yes, I definitely could find myself relaxing more using these techniques’*). Those who answered ‘no’ to question 3 explained that they did not find the rtfMRI-NF procedure enjoyable, and described it as *tiring and confusing*. All but one participant thought the study was good and five participants specifically mentioned that they enjoyed the rtfMRI-NF experience. When the participants were asked what they did not like about the study (question 5), two of the participants responses suggested that the rtfMRI-NF procedure was too difficult for them; ‘*the fact that not one technique for the rocket task would work properly, it was like I was making it up as I went’.* Three of the participants commented that they found the length of the study too long; *‘I did not like being in the scanner for so long’.* Similarly, when asked how the study could be better (question 5), the majority of the responses referred to the difficulty of the rtfMRI-NF procedure and the length of the study; ‘*It does take a lot of time so it could be shorter in some way’*. No responses by the participants were given for question 6 where they were asked to share any additional comments on the study.

1. Thomas DR. A general inductive approach for analyzing qualitative evaluation data. *American Journal of Evaluation* 2006; **27**(2)**:** 237-246.

Appendix

***Interview questions after NF-training session on visit 1-4 (post-scan):***

1. What are your thoughts on the scanning visit
2. What strategy did you use during the rocket NF task?

***Additional questions after visit 4 (post-scan):***

1. Did you find participating in this study useful?
2. Do you think you have learned anything useful? (Y/N)

If yes,

1. Do you think you will apply the strategies that you have learned outside of this study, for example in your daily life?

If no,

1. In your opinion, why do you think you did no learn anything? Is there anything that could have been done to support you in getting more out of this study?
2. What did you think was good about this study?
3. What do you think was not so good about this study?
4. From your personal experience, how could this study been made better for you?
5. Please tell us anything else you would like to share and comment on about your experience of this study

Movement parameters of the excluded study participant.

Table 2. Maxiumum dispersion of movement during the rtfMRI-NF training.

|  |  | **x** | **y** | **z** |
| --- | --- | --- | --- | --- |
| **Session 1** |  | 0.2414 | 0.3541 | 0.1561 |
| **Session 2** |  | 0.3441 | 0.4761 | 1.7122 |
| **Session 3** |  | 0.3247 | 0.1997 | 0.4059 |
| **Session 4** |  | 0.1891 | 0.2681 | 0.8885 |
| **Session 5** |  | 0.3065 | 0.5823 | 1.134 |
| **Session 6** |  | 0.1396 | 0.2459 | 1.6213 |
| **Session 7** |  | 0.2042 | 0.2142 | 0.6176 |
| **Session 8** |  | 0.2806 | 0.3637 | 0.7132 |
| **Session 9** |  | 0.583 | 0.2097 | 3.1313 |
| **Session 10** |  | 0.2043 | 0.3591 | 2.6746 |
| **Session 11** |  | 0.3911 | 0.3223 | 2.165 |
| **Transfer** |  | 0.2362 | 0.4964 | 2.5962 |

Session 1)


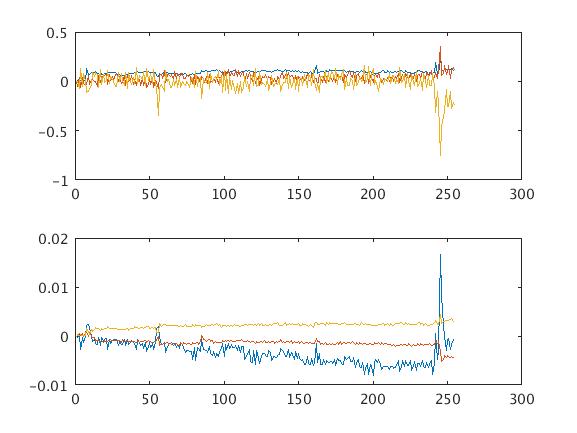


Session 2)


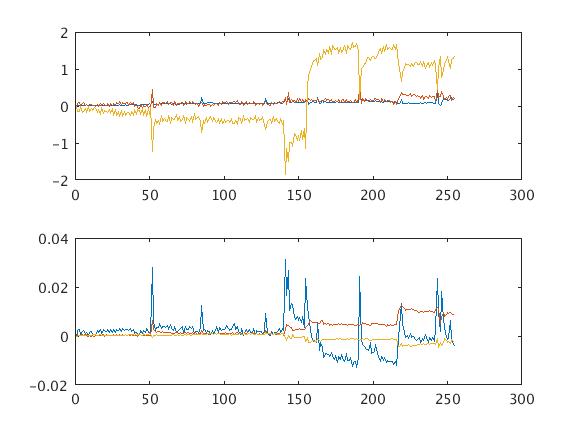


Session 3)


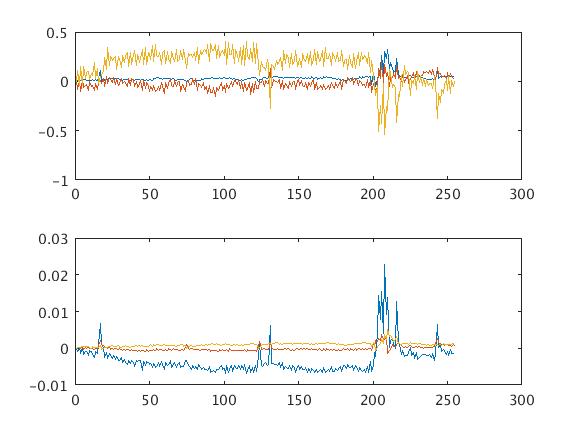


Session 4)


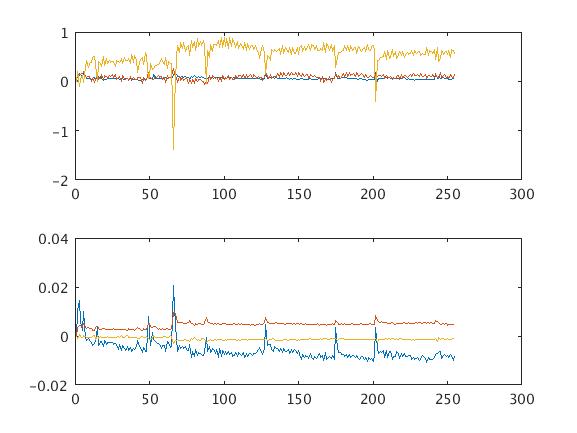


Session 5)


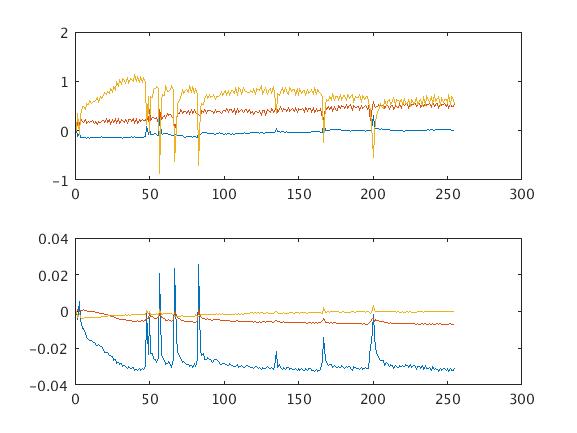


Session 6)


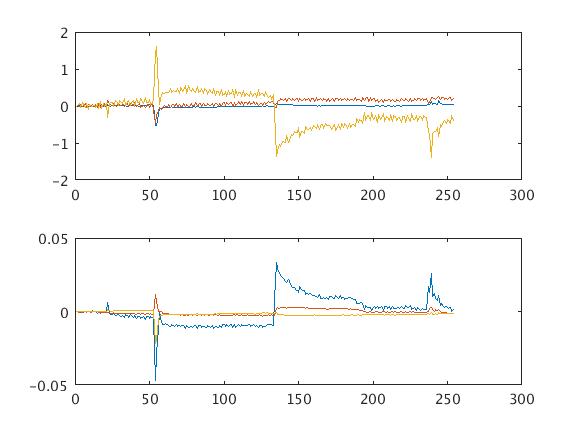


Session 7)


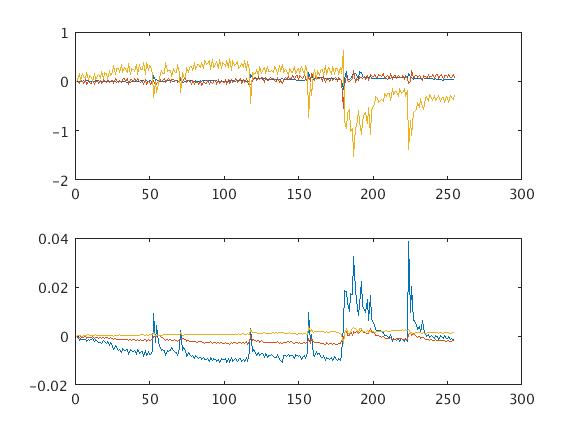


Session 8)


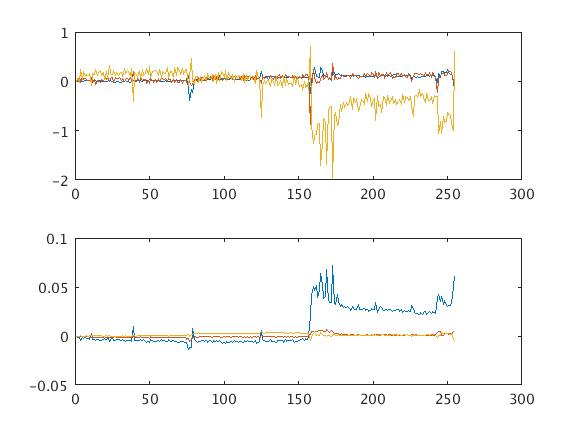


Session 9)


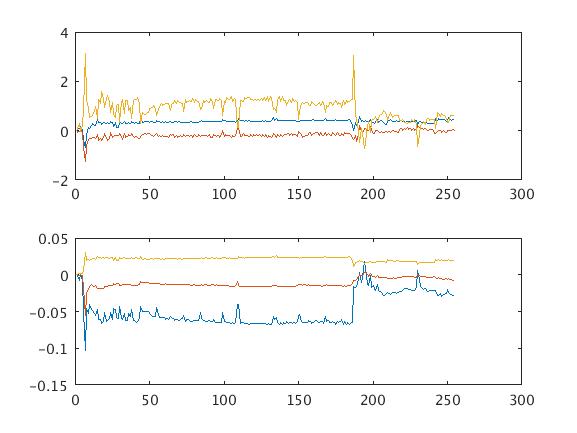


Session 10)


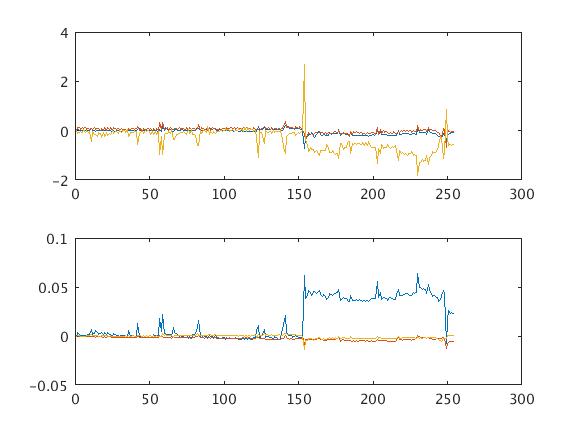


Session 11)


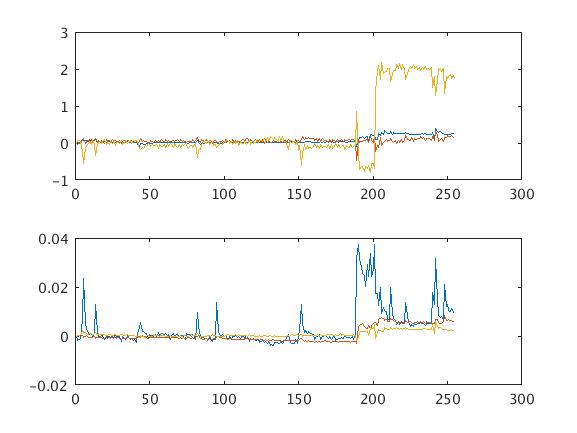


Transfer


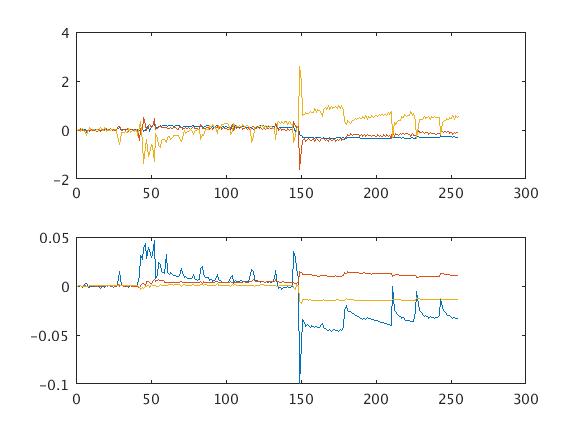

Supplement: Supplementary file 1 — Supplementary material [file 41398_2017_67_MOESM1_ESM.docx]
